# Genetic analysis and QTL mapping of the seed hardness trait in a black common bean (*Phaseolus vulgaris*) recombinant inbred line (RIL) population

**DOI:** 10.1007/s11032-018-0789-y

**Published:** 2018-02-23

**Authors:** K. S. Sandhu, F. M. You, R. L. Conner, P. M. Balasubramanian, Anfu Hou

**Affiliations:** 1 Morden Research and Development Centre, Agriculture and Agri-Food Canada, Morden, MB R6M 1Y5 Canada; 2 Lethbridge Research and Development Centre, Agriculture and Agri-Food Canada, Lethbridge, AB T1J 4B1 Canada

**Keywords:** Cooking quality, Seed hardness, Common bean, QTL mapping, *Phaseolus vulgaris*

## Abstract

Seed hardness trait has a profound impact on cooking time and canning quality in dry beans. This study aims to identify the unknown genetic factors and associated molecular markers to better understand and tag this trait. An F_2:7_ recombinant inbred line (RIL) population was derived from a cross between the hard and soft seeded black bean parents (H68-4 and BK04-001). Eighty-five RILs and the parental lines were grown at two locations in southern Manitoba during years 2014–2016. Seed samples were harvested manually at maturity to test for seed hardness traits. The hydration capacity and stone seed count were estimated by soaking the seeds overnight at room temperature following AACC method 56-35.01. Seed samples from 2016 tests were also cooked to determine effect of seed hardness on cooking quality. For mapping of genomic regions contributing to the traits, the RIL population was genotyped using the genotype by sequencing (GBS) approach. The QTL mapping revealed that in addition to the major QTL on chromosome 7 at a genomic location previously reported to affect seed-hydration, two novel QTL with significant effects were also detected on chromosomes 1 and 2. In addition, a major QTL affecting the visual appeal of cooked bean was mapped on chromosome 4. This multi-year-site study shows that despite large environmental effects, seed hardness is an oligo-genic and highly heritable trait, which is inherited independently of the cooking quality scored as visual appeal of cooked beans. The identification of the QTLs and development of SNP markers associated with seed hardness can be applied for common bean variety improvement and genetic exploitation of these traits.

**Electronic supplementary material:**

The online version of this article (10.1007/s11032-018-0789-y) contains supplementary material, which is available to authorized users.

## Introduction

Legumes contribute an average of 2.5 and 7.5% of total protein intake in developed and developing countries, respectively. In 28 countries belonging to the latter category, this figure is greater than 10% (Akibode 2011). The type of legumes consumed, however, varies from region to region. In sub-Saharan Africa, Latin America, and the Caribbean, dry beans (*Phaseolus vulgaris* L.) are the major legumes produced and consumed. In 2006–2008, worldwide dry bean production reached 15 million ha with a harvest of 10.65 million t (Akibode and Meridia 2011). In Canada, dry bean is an important rotation and cash crop, planted on 119,000 ha in 2016. Canada is also the world’s fifth-largest exporter of dry bean with an annual production of 229,000 t in 2014 (Statistics Canada 2016). In the near future, with greater emphasis on sustainable agriculture and dietary diversification, the importance of dry bean cultivation is likely to increase. Moreover, the effect of climate change on crop productivity could favor dry bean production in the northern hemisphere, including Canada (Ramirez-Cabral et al. 2016).

Cooking quality is an important factor for bean consumers worldwide. Beans are consumed after traditional cooking or in canned form. Therefore, both cooking and canning quality attributes are important for the success of bean varieties (Castellanos et al. 1997; Kelly and Cichy 2013). Traditional cooking and canning quality attributes of dry bean seed include cooking time, absence of stone seeds, hydration capacity, texture, and appearance etc. Like most legumes, bean seeds are prone to seed hardness. Seed hardness in legumes refers to the phenomenon requiring extended cooking time to allow softening to a desired texture. Hard seeds that do not imbibe any water during hydration or cooking are also known as “stone seeds”. Seed hardness affects bean seed cooking time and the hydration process in canning (Aguilera and Stanley 1985; McWatters et al. 1987). In addition, seed hardness trait has other negative impacts such as increased cost of consumption, loss of nutritional quality, canning quality, and uneven germination when seed is planted for field production (Stanley 1992).

Seed hardness is heritable trait while it is also affected by environmental factors during production and even under seed storage conditions (Argel and Paton 1999). The genetic factors affecting seed hardness are not well understood and could vary from a simply inherited trait such as *ASPER* (*Asp*) gene to an unknown number of major or minor genes. The *Asp* gene is associated with seed coat luster without affecting the color. Genotypes which lack *Asp* gene display dull/matte/opaque seed coats (Bassett 1996). Seed coat shininess has been associated with a low rate of water uptake in genotypes (Bushey et al. 2000). The impact of seed coat luster on water uptake was shown through the use of isogenic lines that differed only at the *Asp* locus (Konzen and Tsai 2014). However, in the same study, it was also shown that *Asp* was not the sole determinant of water uptake rates in black bean lines of different genetic origins. Water uptake of a variety is one of the most important criteria for consumers and the misconception that shiny seeded varieties are always poor for water imbibition has affected consumer preference. Breeding for shiny seeded varieties is beneficial on other accounts. Shiny seeds probably handle the environmental and storage stresses better than dull seeds (Diamant et al. 1989). Shiny-seeded varieties display a thicker palisade cell layer under microscope (Konzen and Tsai 2014). Shiny-seeded varieties are also known to contain more anthocyanins in their seed coats; however, this trait does not seem to have an impact on the anthocyanin content of the canned beans (Cichy et al. 2014). The *Asp* locus has been genetically mapped to chr 7 in several studies (Pérez-Vega et al. 2010; Cichy et al. 2014). However, the underlying gene and its mechanism have not been identified.

The environmental factors impact seed hardness through the phenomenon known as the hard-to-cook (HTC) defect. This defect occurs when legume seeds are maintained under adverse storage conditions, such as high temperature and high humidity. The mechanism of the HTC induction is still not fully understood (Liu et al. 1992). Many theories have been postulated to explain the origins of the HTC defect in legume seeds, the most documented among these is pectin-cation-phytate-phytase theory (Galiotou-Panayoutu et al. 2007; Kinyanjui et al. 2015). This theory postulates that the activity of phytase in seeds, under adverse conditions, leads to degradation of phytic acid causing the release of metal cations. These metal cations, chiefly Ca++, migrate to intercellular spaces to bind pectins and thereby rendering them as insoluble pectates. Although environment induced, the HTC phenomenon itself is not independent of genetic influences, since some varieties are more prone to HTC defect than others (Shiga et al. 2004).

It is possible that similar genetic factors are involved in seed hardness under normal conditions and hard-to-cook defect induced by adverse environment. Therefore, to develop superior varieties, it is vital to understand the underlying genetic factors that render varieties susceptible to seed hardness and/or the cause of the HTC defect. Testing for the seed hardness trait is not practical during the early breeding stages as it requires a large seed samples for effective determination. Moreover, the trait is highly influenced by environment, and therefore, multiple biological replicates are required to make an accurate assessment of the trait. Again, this is generally not feasible in early stages of breeding process. This difficulty makes seed hardness an ideal trait for molecular marker tagging and assisted breeding selection. Unfortunately, apart from *Asp* gene, there are no known molecular markers associated with the seed hardness trait in dry beans. However, QTLs have been reported for the calcium and magnesium content of the seed coat and water absorption (Pérez-Vega et al. 2012; Casañas et al. 2013). It is possible that these may be correlated to the incidence of stone seeds as this also agrees with the mechanism of HTC in pectin-cation-phytate-phytase theory. It is likely that more unexploited variation for this trait is present in germplasm collections. Germplasm of diverse origins should be used in QTL studies to identify genes that impact this trait. Therefore, a black bean recombinant inbred population was generated by crossing parents different for seed hardness trait.

The main objective of this study was to map the QTL for seed hardness traits and develop molecular markers for use in breeding. Multi-environmental field data was used to map three significant QTL for seed hardness and a QTL for the appearance of cooked beans. Coincidentally, several QTLs for seed weight and seed yield were also mapped.

## Material and methods

### Plant material and field experiment conditions

A total of 114 F_2:7_ RILs were derived through a single-seed decent method from a cross between black bean lines BK004-001 and H68-4. BK04-001 and H68-4 were breeding lines developed at the Morden Research and Development Centre (MRDC), Morden, Manitoba, Canada. BK04-001 showed a low incidence of stone seeds and H68-4 was identified with the highest stone seed count among the breeding lines screened in the year of selection (2010). The initial reciprocal F_1_ cross-pollination was made in 2011 in the greenhouse at MRDC. Seed was scarified before planting to encourage the uniform germination during each generation of the RIL advance. Randomly selected 85 RILs and two parents were grown in the field at Morden (49.1923°N, 98.0977°W, elevation 297.50 m) and Carman (49.5086°N, 98.0017°W, elevation 268 m) sites for 3 years (2014–2016) using randomized complete block design (RCBD) with three replications. Average growing season minimum and maximum temperatures in Morden were 12.4, 13.5, 13.4, 23.9, 25.7, and 24.6 °C, respectively, during the 3 years of the study. Average growing season minimum and maximum temperatures in Carman were 10.8, 11.7, 11.9, 23.6, 25.7, and 24.3 °C, respectively, during the 3 years of the study. Total precipitation for 2014, 2015, and 2016, respectively, during the growing seasons was 243.9, 171.5, and 364.6 mm in Morden and 291.7, 253, and 252.6 mm in Carman in the 3 years, respectively. However, data from the Carman trial in 2015 was excluded from analysis due to extensive flooding damage to the trial in early July.

### Phenotyping

Seeds for phenotypic analyses were harvested at maturity. Seeds were harvested with a combine in 2014, but manually harvested in 2015 and 2016. Manual harvest was used to avoid any potential damage to the seed coats, which was suspected to have a major impact on the traits under study. In 2016 at the Carman site, seeds were harvested twice at the interval of 2 weeks to study the effect of timing of harvest after full maturity. Seed hardness was measured as two negatively correlated traits, namely stone seed percentage (SSP) and hydration capacity (HC) following AACC method 56-35.01 (AACC International 2007). HC was defined as the ratio of hydrated seed weight to dry seed weight. Stone seeds are the seeds, which stayed completely un-hydrated after 16 h soak in water (at 22 ± 2 °C). These traits were measured as follows: field harvested seeds were air-dried to approximately 10% moisture level before phenotyping. A random sample of 100 intact-looking seeds from each plot were weighed and soaked in water for 16 h at room temperature (22 °C). After 16 h, seeds were drained of excess water, strained, and weighed to calculate HC (ratio of hydrated seed wt. to dry wt.). Numbers of stone seeds were also counted to calculate the stone seed percentage. Three components of color, L*, a*, and b*, were measured using CM-5 Spectrophotometer (Konica Minolta INC, Japan). Parameter L* is the lightness component and can range in values between 0 (black) and 100 (white). Parameter a* varies between green to red and parameter b* from blue to yellow, with values ranging from − 120 to + 120. If two seed sample shares the same values of L*, a*, and b*, that indicates the samples will be perceived as having exactly same color by the human eye. Color loss as a result of cooking on the beans also was evaluated. Due to the lack of a canning facility, we used an alternative method for assessing cooking quality analysis. Samples from 2016 harvest were cooked for 20 min in a water bath at 96 °C. Cooking was done after a 16-h soak at room temperature. Due to the unevenness of color loss in cooked samples and presence of stone seeds, it was not feasible to get an accurate reading of seed color using spectrophotometer. Therefore, the cooked seeds were visually scored for color loss and visual appeal. A score between 0 and 5 was assigned to each sample, in which low score indicated good visual appeal and better color retention (including uniformity of color) and a high score indicates a poor visual appeal and low color retention after cooking. Two agronomic traits were also measured; total seed yield from the field plots was converted to kilograms per hectare, and weight of 100 randomly selected dry seeds from the field samples was measured to obtain 100-seed weight (SW) (g). In addition, the flowering dates were recorded as the days from planting to 50% plants with at least one flower; the maturity date was recorded as the days from planting to 90% plants matured for harvest; growth habit was recorded following the description of van Schoonhoven and Pastor-Corrales (1987).

### Scanning electron microscope

Seed-coat microstructure of the parental lines was studied using the Quanta 650 FEG Environmental Scanning Electron Microscope (FEI, Hillsboro, Oregon) at the Engineering Department, University of Manitoba (Winnipeg, Canada). The seeds were sliced with a glass knife on a microtome. The sliced/split seeds and cross-sections were mounted on scanning electron microscope stubs (aluminum) with the help of a double-sided Carbon Tape. Later, the samples were coated with a thin (10–15 nm) layer of Au-Pd using a Denton Desk II Sputter Coater. The coated samples were viewed in high vacuum mode with ETD detector (Everhart Thornley Detector).

### Genotyping by sequencing and linkage map construction

A genotype-by-sequencing (GBS) approach was used to generate SNP markers for the RIL population. The 114 RILs and parents were grown in the greenhouse at MRDC. Young leaves were used to isolate genomic DNA using DNeasy Plant Mini kit (QIAGEN, Valencia, CA). Isolated DNA samples were checked for quality using agarose gel electrophoresis and then sent to Genome Diversity Facility (Cornell University, Ithaca, NY) for sequencing. The GBS technique is described in detail by Elshire et al. (2011). Briefly, genomic DNA was fragmented using a type II methylation sensitive restriction endonuclease enzyme, *Ape*KI that recognizes the cut site CWGC. The fragmented DNA was ligated with barcoded adapters and was amplified using appropriate primers in a polymerase chain reaction (PCR). The amplified fragments were then sequenced on an Illumina Hiseq 2500 instrument (Illumina Inc., San Diego, CA). The raw sequence data were then filtered for quality and aligned with the *P. vulgaris* reference genome (Phytozome v11, Schmutz et al. 2014) using the Burrows-Wheeler Alignment tool (BWA V0.78-r455) integrated in the GBS analysis pipeline as described in Glaubitz et al. (2013).

A total of 80,398 single-nucleotide polymorphisms (SNPs) were identified from the 114 RILs and two parents with a minor allele frequency (MAF) > 0.01 and missing data rate per site < 90%. A total of 3115 SNPs were retained after removing those with a missing data rate per site > 10% for downstream analyses. The redundant SNPs which had strong linkage disequilibrium (LD) were further removed and only one SNP marker was retained in the same LD block. This resulted in the final 619 SNP markers for the linkage map construction. Due to significant segregation distortion from the expected 1:1 (*p* > 0.05), eight SNPs were also excluded from the genetic mapping. Consequently, a genetic linkage map was constructed from the data of 611 SNPs in 114 RILs (including the 85 RILs used in phenotypic analysis) using IciMapping V 4.1 (Li et al. 2007). To construct the linkage map, SNP markers were grouped based on LOD score of 4.0 and ordered using REcombination Counting and ORDering (RECORD) and COUNT algorithm (Van Os et al. 2005). Genetic map distance was estimated in centi-Morgan (cM) based on Kosambi mapping function. Linkage groups were oriented and assigned to the chromosomes using anchoring markers from the *P. vulgaris* consensus map (Galeano et al. 2011), and matching SNP coordinates from GBS data with the genome sequence information (DOEJGI, www.phytozome.net). A few SNP markers closely linked to seed hardness traits were also converted to dCAPS markers (Neff et al. 1998) for marker-assisted selection purposes. A graphical representation of genetic map was constructed using MapChart (Voorrips 2002). For the QTL effect and interaction plots, the phenotypic values were plotted against the genotypes at one and two linked markers, respectively. The QTL effect and interaction plots were generated using “plotPXG” and “effectplot” function of the “qtl” package in R (Broman et al. 2003).

### Field experiment data analyses

To reduce the heterogeneity of variance and meet the normality assumption, stone seed percentage data were converted to proportion and transformed using log transformation. The data for other traits were used in their original form as they met the assumptions of normality (W-test stat > 0.90). Analysis of variance for each year and site was performed separately. For calculation of heritability parameter, all effects were considered random. The basic model used was as follows:

$$ {Y}_{ij}=\upmu +{\beta}_i+{\tau}_j+{\varepsilon}_{ij} $$

where μ is the mean, *β*_*i*_ is the block effect of *i*th block, and *τ*_*j*_ is the treatment effect of the *j*th treatment. *ε*_*ij*_ is the error, following a normal distribution *N*(0, ${\sigma}_e^2$). ${\sigma}_e^2$ is the error variance. Broad-sense heritability was calculated on the entry mean basis as follows:

$$ H=\frac{\sigma_g^2}{\sigma_g^2+\frac{\sigma_e^2}{r}} $$

where *σ*^2^_*g*_ and *σ*^2^_*e*_ are the genetic and error variances, respectively, and *r* is the number of replications. Statistical calculations were performed using the META-R (Multi-Environment Trial Analysis with R for windows) (Alvarado et al. 2015). Best linear unbiased estimates (BLUEs) were calculated for various traits using META-R. BLUEs were calculated using ordinary mean squares and considering all effects as fixed. Combined data from Morden and Carman sites in 2016 were used to calculate Pearson’s correlation coefficients between traits using “*rcorr*” function in “Hmisc” package in R (R Development core team 2008).

### QTL analysis

Quantitative trait loci (QTL) analysis was performed on BLUEs for all traits. For the stone seed trait, BLUEs from both untransformed and transformed data were used for QTL mapping. The results were similar; therefore, only the results from the original untransformed data were adopted. For QTL mapping, inclusive composite interval mapping (ICIM), a mapping function that considers all markers simultaneously to compute stepwise regression of the markers was used (Li et al. 2007). To test the statistical significance of QTL candidates, the logarithm of odds (LOD) score was estimated through 1000 permutation tests (Churchill and Doerge 1994) and a type 1 error rate of α ≤ 0.05. Only QTL with LOD score above the threshold was retained. The genotypic variation explained by each QTL was calculated from the ratio: *R*^2^/*H* where *R*^2^ is the percent phenotypic variation explained by the QTL and *H* is the broad-sense heritability estimate for the trait.

## Results

The parental lines, BK04-001 and H68-4 of the RIL, had similar growth habits (type I) and required the same number of days to flowering (48–51) and to reach maturity (95–100). However, they displayed significant differences in seed hardness traits (Table 1). Two parents differed in their seed coat lustres, with shiny for H68-4 and opaque/matte for BK04-001. Their seed hardness traits were also significantly (*P* < 0.05) different in all environments while they did not produce significant differences in agronomic or color traits in all the cases (Table 1). Among the RILs, significant (*P* < 0.05) differences were observed for all traits. Transgressive segregation was also observed for all the traits, indicating that the parents were genetically diverse for these traits (Table 1). Seed hardness was measured in terms of SSP and HC. Although there was an inverse relationship between these two traits, they were also complementary to each other as HC measurement took into account all seeds, including fully and partially hydrated while SSP was only based on completely un-hydrated seeds (Supplementary Fig. 1). In 2014, when trials were harvested with a combine harvester, the SSP was lower than that in years 2015–2016. As indicated from the SSP means of the RILs, SSP values were slightly higher in the Carman (CA) trials as compared to Morden (MO) traits (Table 1). In terms of harvesting time in 2016, the seeds from the second harvest had fewer stone seeds than those from the first, although their SSP range and heritability estimates were similar (Table 1).

**Table 1:** Means and ranges of seed traits for the parents and RILs, and broad-sense heritability estimates in RILs based on three replications grown at two locations during 2014–2016 in Manitoba, Canada

Traits	Years	Site	Parents		*P* value	RILs		
			BK004	H68-4		Mean	Range	Heritability (%)^b^
Stone seed percentage (SSP)	2014	MO	0.5 ± 0.70	25.6 ± 4.04	**	9.45 ± 12.2	0–51	90.32
		CA	0.0 ± 0.00	24.3 ± 2.10	**	11.55 ± 15.5	0–64	95.24
	2015	MO	0.7 ± 1.15	23.6 ± 2.51	**	14.3 ± 21.9	0–95	90.29
		CA	–	–		–	–	–
	2016	MO	10.7 ± 8.50	37.3 ± 9.70	**	47.9 ± 32.0	0–100	94.23
		CA	21 ± 8.50	64.5 ± 27.60	**	51.9 ± 27.5	0–99	93.16
		H2CA^a^	2.0 ± 2.64	55.0 ± 8.70	**	27.4 ± 25.3	0–96	92.74
Hydration capacity (HC)	2014	MO	2.24 ± 0.14	1.93 ± 0.04	**	2.14 ± 0.23	1.49–2.86	94.85
		CA	2.21 ± 0.06	1.89 ± 0.06	**	2.05-0.25	1.44–2.81	97.10
	2015	MO	2.24 ± 0.05	1.74 ± 0.14	**	1.97 ± 0.32	1.21–2.48	90.43
		CA	–	–	–	–	–	–
	2016	MO	1.94 ± 0.14	1.61 ± 0.11	**	1.65 ± 0.34	1.16–2.41	95.02
		CA	1.89 ± 0.16	1.36 ± 0.07	**	1.58 ± 0.29	1.12–2.27	94.44
		H2CA	2.15 ± 0.09	1.46 ± 0.06	**	1.81 ± 0.30	1.10–2.32	94.93
L*	2016	MO	15.23 ± 0.53	16.58 ± 0.64	*	15.81 ± 0.64	14.25–17.87	29.71
		CA	15.38 ± 0.91	15.54 ± 0.29	NS	14.94–0.63	13.27–16.85	46.93
a*	2016	MO	0.38 ± 0.05	0.47 ± 0.04	NS	0.43 ± 0.11	0.14–0.82	72.69
		CA	0.38 ± 0.04	0.42 ± 0.03	NS	0.43 ± 0.10	0.15–0.72	81.10
b*	2016	MO	0.33 ± 0.12	0.82 ± 0.45	**	0.56 ± 0.27	− 0.29–1.52	56.28
		CA	0.24 ± 0.20	0.62 ± 0.02	*	0.55 ± 0.19	− 0.01–1.14	80.64
Visual score (VSC)	2016	MO	3.83 ± 0.28	1.83 ± 0.28	**	2.9 ± 0.72	1.0–4.5	70.33
		CA	4.0 ± 0.00	2.0 ± 0.00	**	3.12 ± 0.8	1.0–5.0	82.86
		H2CA	3.83 ± 0.28	2 ± 0.00	**	2.96 ± 0.97	1.0–4.5	77.19
100 seed wt. (SW)	2014	MO	21.69 ± 0.61	19.75 ± 0.87	NS	20.03 ± 1.58	16.22–24.59	88.46
		CA	22.76 ± 0.36	20.84 ± 0.25	**	21.74 ± 1.39	16.66–26.64	92.77
	2015	MO	20.87 ± 0.84	19.39 ± 0.18	*	20.10 ± 1.33	17.17–23.79	90.77
		CA	–	–	–	–	–	–
	2016	MO	23.73 ± 1.09	21.95 ± 0.75	NS	22.2 ± 1.58	17.5–26.64	85.33
		CA	23.24 ± 0.39	23.09 ± 0.13	NS	22.49 ± 1.32	19.1–26.31	82.14
		H2CA	22.73 ± 0.24	23.01 ± 0.63	NS	22.3–1.35	18.1–25.53	82.37
Seed yield (SY)	2014	MO	1633 ± 285	2398 ± 158	*	1452 ± 434	480–2603	65.76
		CA	1894 ± 618	2403 ± 243	NS	2070 ± 397	592–3235	77.06
	2015	MO	2430 ± 456	2632 ± 468	NS	1795 ± 652	125–4042	67.58
		CA	–	–		–	–	–
	2016	MO	1692 ± 550	2208 ± 683	NS	1476 ± 475	390–2778	77.46

*MO* Morden, *CA* Carman

^a^H2CA indicates the data are based on seed samples collected from second harvest at the Carman site

^b^Heritability values of SSP were calculated from the transformed values

As expected, a similar trend was evident for HC, given the inverse and proportional relation of this trait with SSP. Heritability estimates for SSP and HC were greater than 90% in all environments, indicating strong genetic basis for the observed variability. Color parameters were only measured in 2016 in seed samples from the Morden trial and the first harvest of Carman trial (CA16). Among the color parameters, L* had the lowest heritability, and a* had the highest (Table 1). There were also significant differences among sites for heritability of color parameters. Among agronomic traits, the heritability of 100 seed weight (SW) was higher than that of seed yield (SY).

Pearson’ correlation coefficients indicate that SSP was significantly correlated with all traits except L* (Table 2). HC was also correlated to all traits except L* and visual appearance score (VSC). L* was not correlated with a*; however, both L* and a* were significantly correlated with b*. As expected, SY was significantly correlated with SW. VSC was correlated with SSP, a*, and SW. These correlations, positive or negative, suggest linkages of corresponding trait QTL on the genetic map.

**Table 2:** Pearson correlation coefficients between various traits in the RIL population

	HC	L*	a*	b*	SY	SW	VSC
tSSP	− 0.93***	0.0074	0.32***	0.21***	0.33***	0.15***	0.13**
HC		0.039	− 0.38***	− 0.23***	− 0.35***	− 0.21***	− 0.087
L*			− 0.038	0.20***	0.16*	0.11*	0.007
a*				0.49***	0.11	− 0.031	0.15***
b*					0.20**	0.041	− 0.10*
SY						0.38***	− 0.033
SW							0.15***

*tSSP* transformed stone seed proportion, *HC* hydration capacity, *SY* seed yield, *VSC* visual score of cooked seed appearance, *SW* 100-seed weight

*, **, and *** represent the statistical significance at 0.05, 0.01, and 0.001 probability level. Correlations were calculated based on the data from Morden and Carman (first harvest data) in 2016

**Fig. 1:**
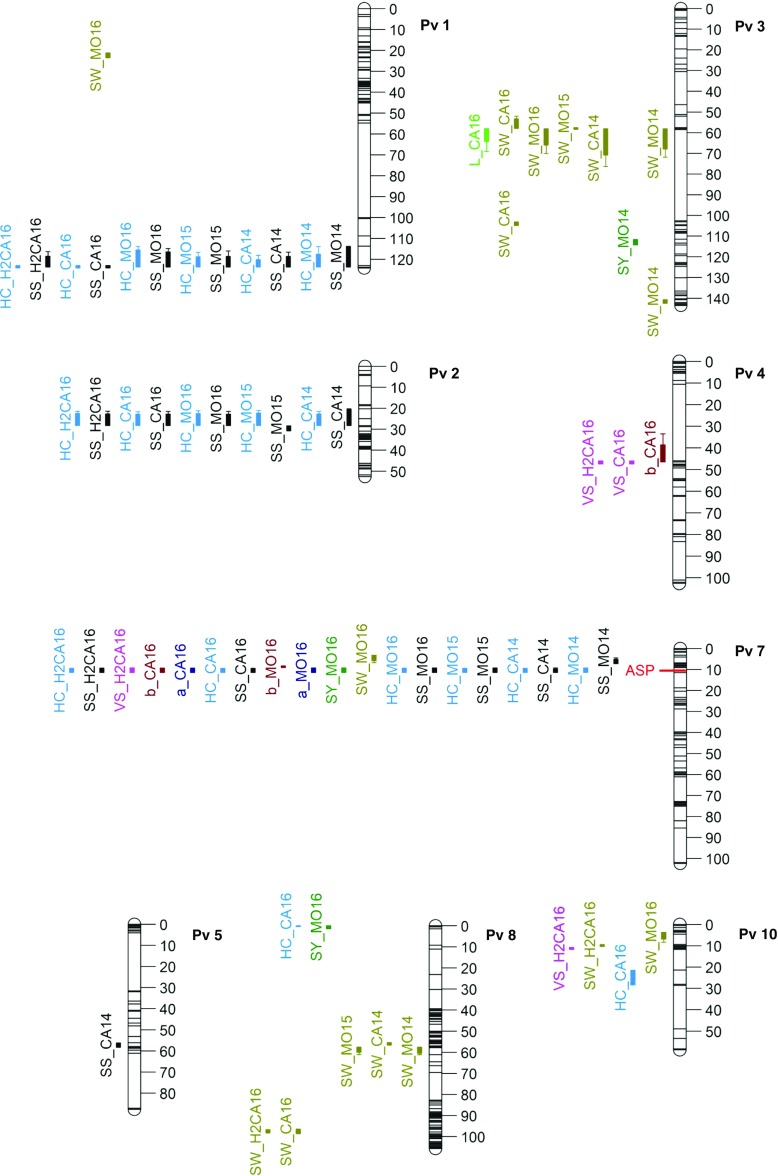
Linkage map of identified QTL for all traits. QTL are depicted left of the chromosomes with solid bars indicating 1-LOD interval and outer whiskers indicating 2-LOD interval. The QTL labels are derived by joining trait name abbreviation and site with an underscore

The genetic map was constructed based on the mapping data generated from 114 RILs and 611 SNP markers (Fig. 1). One phenotypic marker, *Asp* (asper) based on seed coat luster (Lamprecht 1940), was also scored. In addition, some InDel markers (Moghaddam et al. 2014) and derived cleaved amplified polymorphic sequence (dCAPS) markers were also developed to fill in the gaps between two SNP markers to reduce the size of intervals surrounding certain QTL. These SNP and dCAPS markers were mapped to a total of 114 bins. Total map length was 1024 cM with markers present on all 11 chromosomes (Fig. 1 shows only chromosomes in which QTL were mapped). A total of 27 unique QTL for eight traits were mapped in this study (Fig. 1). Table 3 provides the summary of QTL analysis results.

**Table 3:**
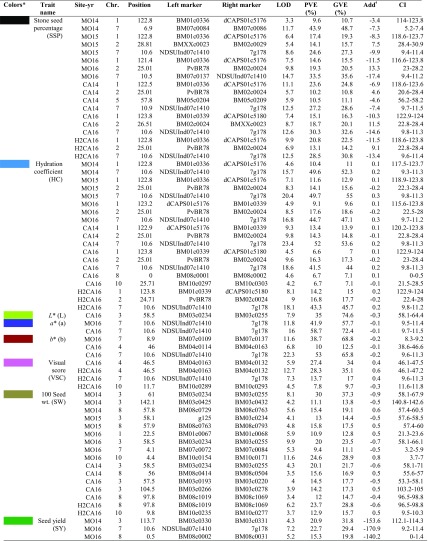
Chromosome locations and effects of all significant QTL discovered in this study using the RIL population derived from BK04-001/H68-4 and phentoyped at two sites during 2014–2016 in Manitoba, Canada

*Colors correspond to the QTL in Fig. 1

^ǂ^Additive effect values indicate the change in trait value obtained by replacing H68-4 allele with BK04-001 allele

*PVE* phenotypic variance explained, *GVE* genetic variance explained, *CI* confidence interval

**Fig. 2:**
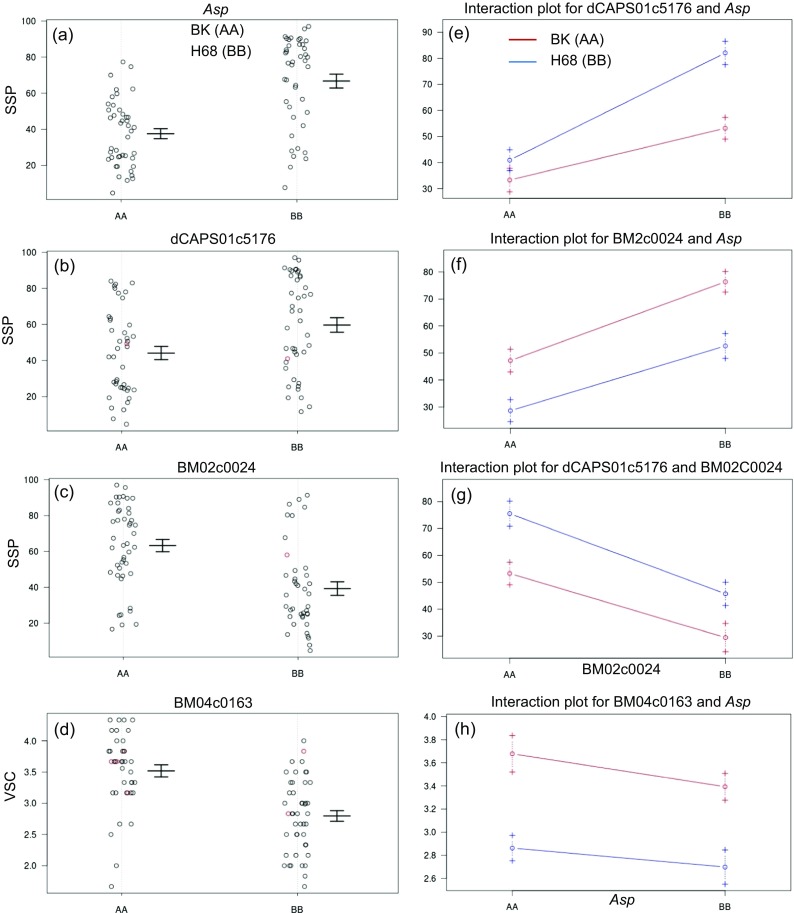
QTL effects (**a**–**d**) and interactions (**e**–**h**) estimated from the first-harvest seeds at Carman site in 2016. Error bars indicate SE

QTL affecting seed hardness traits, SSP and HC, were mapped onto chromosomes (chrs) 1, 2, 5, 7, 8, and 10. Among these, QTL on chrs 1, 2, and 7 were detected in all environments except in Morden 2014. In contrast, QTL on chrs 5, 8, and 10 were only detected in single environments. The QTL on chr 7 was mapped in the vicinity of *Asp* gene, as reported by Cichy et al. (2014), with an interval of 326.6 kb. Phenotypic variations explained (PVE %) by the major QTL on chr 7 for SSP ranged from 28.5 to 43.9% in different trials (Table 3). The *Asp* phenotypic marker and left-flanking marker NDSUInd07c1410 also shared the same bin, suggesting a tight linkage between seed coat luster and *Asp* for the seed hardness. The QTL for SSP and HC on chr 1 resided at the distal lower arm, with an interval (smallest) of 13.6 kb. The PVE explained by this QTL ranged from 9.6 to 23.6%. The location of QTL on chr 2 was located at the upper arm with an interval of 2 Mbp, and the PVE accounted for by the QTL ranged from 10.2 to 19.3%, respectively. QTLs on chromosomes 1 and 7 were contributed by the hard-seeded H68-4 parent and the QTL on chr 2 was contributed by the soft-seeded parent BK04-001. In addition, QTL for SW were detected on chrs 1, 3, 8, and 10. QTL for SW also were contributed by both parents, as indicated by transgression in the range of values (Table 1). QTL increasing SW on chr 3 was contributed by H68-4, and QTL on chrs 8 and 10 were contributed by BK04-001. In contrast, all three SY QTL for increasing yield were contributed by H68-4. Interestingly, a major VSC QTL that accounted for 28.3% of PVE was detected on chr 4 in the 2016 Carman trial. Similar VSC QTL peaks were also detected in Morden 2016 trial, but their LOD scores were below the detectable threshold. QTL for color parameters, b*, were also detected on chrs 4 and 7. Figure 2 shows graphically the QTL effects and interactions for the 2016 Carman test for three QTL affecting SSP trait and the major QTL on chr 4 affecting VSC. The effects and patterns were similar for the Morden 2016 test. Supplementary Fig. 2 reports the QTL effects and their interactions for data collected from the second harvest of the 2016 Carman test. As seen in the figure, the QTL effects were not affected by the harvest time.

In order to determine the role of seed coat on the production of stone seeds, scanning electron microscope (SEM) analysis of BK04-001 and H68-4 seeds was conducted (Supplementary Fig. 3). The shiny seeds of H68-4 displayed smoother seed coat surface as compared to the BK04-001 seeds which had a rougher surface. No prominent wax layer was observed on the seed coat of either parent. Thickness of the palisade layers was measured and there were no significant differences among the parents (BK04-001 = 29.69 μm ± 0.31 SE, H68-1 = 28.66 μm ± 0.28 SE; *P* = 0.1498).

## Discussion

Stone seeds have been identified as a major production issue under short season growing conditions in the bean breeding program at MRDC. Stone seeds were a serious concern in all bean market classes, especially black beans. Furthermore, the trait was also shown to be highly affected by environment, hampering selection efficiency in breeding. The aim of this multi-year study was to find stable genetic factors mainly affecting seed hardness, and therefore cooking quality in black beans. Using a RIL population, several QTL were mapped for seed quality traits as potential targets for selection in breeding programs. The mean number of stone seeds was affected by factors such as year, location and harvesting time (Table 1). Mean SSP values were always higher at the Carman site, as was SW. Although effect of harvesting time was only studied in 2016 at one location, those findings match the general experience over the years at MRDC. This indicates a strong role of the local environment at maturity in generating stone seeds. However, the heritability estimates and QTL analyses indicate that regardless of sites, the genetic control of SSP is still stable and strong (Tables 1 and 3).

There was a small but significant positive correlation of SSP with SY and SW in 2016. This was also evident in the QTL mapping results, where SY and SW QTL are co-localized with SSP QTL on chr 7 (Fig. 1). This indicates a positive association between these traits due to close linkage or pleiotropy, which requires further investigation. The correlation of SSP and HC with VSC was 0.13 and − 0.087, respectively. Only one out of three VSC QTL was co-localized with SS and HC QTLs (Fig. 1). The VSC QTL on chr 7 linked with *Asp* explained 13.7% of PVE indicating that seed coat luster had only a small effect on the cooked bean appearance as previously reported by Cichy et al. (2014).

QTL analysis indicated that a gene at/near the *Asp* locus plays a major role in stone seed production and hydration capacity. A QTL at this location was also identified for HC by Cichy et al. (2014). However, no QTL at this location was identified by Pérez-Vega et al. (2010) even though the RIL parents were segregating for seed coat luster. This suggests that a closely linked gene to *Asp* may be responsible for the effect on HC. In addition, two novel and stable QTL were identified in the RIL population for SSP and HC. For SSP, the QTL on chrs 1 and 2 together explained more of the variation than the major QTL on chr 7 (Table 3 and Fig. 2a–c). The effect of the chr 2 QTL was completely additive to the QTL on chr 7 (Fig. 2f). However, the effect of chr 1 QTL was dependent on presence of the chr 7 QTL (Fig. 2e). As seen in the Supplementary Fig. 2, the QTL effects were stable; however, there were some shifts in the interaction patterns between the QTL. The VSC QTL, however, remained independent of *Asp*.

A direct role of *Asp* for the seed hardness trait cannot be ruled out. It was reported that homozygous lines for the recessive *asp* had a rougher cell surface (Beninger and Hosfield 2000; Konzen and Tsai 2014). Theoretically, a rougher seed coat would increase the contact surface and therefore increase water uptake rate during the soaking treatment. The SEM analysis in this study also confirmed differences in surface of seed coats between shiny and matte seeds as previously reported (Beninger and Hosfield 2000; Konzen and Tsai 2014). Those studies also reported differences in thickness of palisade cell layer between shiny and matte lines. However, this was not observed in the current study likely due to the use of non-isogenic lines for SEM analysis. The SEM analysis of BK04-001 and H68-4 parents also did not indicate presence of epicuticular wax layer in either line (Supplementary Fig. 3). Based on the available genomic sequence, both *Asp* and chr 7 QTL were mapped over a 326.6 kbp region. This region contains 41 genes (*P. vulgaris* genome V1.0, www.legnumeinfo.org). In future, the genetic and phenotypic characterization of recombinants in the *Asp* region can be used to determine if the gene underlying *Asp* phenotype and the major QTL are the same. Breeding programs use mostly an elite and narrow genetic base. This usually results in low genetic diversity between the parents resulting in uneven marker density during genetic mapping. The QTL region on chr 2 spans multiple Mbs due to a lack of SNP markers in that region. The QTL region of chr 1 is small enough to suggest some candidate genes. The minimum QTL interval (mapped in 2016 Carman test) contains only the two genes, Phvul.001G264300 and Phvul.001G264400. In *Arabidopsis*, Phvul.001G264300 is homologous to AT2G32000.1 (DNA topoisomerase, type IA, core) and Phvul.001G264400 is homologous to AT2G31980.1 (PHYTOCYSTATIN 2). Among those two genes, Phvul.001G264400 shows exclusive and high level of expression in seed tissues (http://plantgrn.noble.org/PvGEA/SearchVisual.jsp, O’Rourke et al. 2014). Gene sequencing from both parents revealed a mutation in the 3′ end of the gene (Supplementary Fig. 4). This gene is therefore a good candidate for the chr 1 QTL.

In conclusion, the seed hardness trait in black beans is an oligogenic trait that is significantly influenced by the environment. Screening diverse germplasm can help identify the factors affecting seed quality traits. While the environmental factors that impact stone seed production are still unknown, the incidence of the stone seed trait can be improved by using marker-assisted selection. In addition, the cooked appearance of black beans was largely inherited independently from the seed coat luster (*Asp*) and the QTL for hydration capacity. This suggests only a limited trade-off between various seed quality traits.

## Electronic supplementary material

Supplementary Fig. 1 (DOCX 213 kb)

Supplementary Fig. 2 (DOCX 884 kb)

Supplementary Fig. 3 (DOCX 5410 kb)

Supplementary Fig. 4 (DOCX 15 kb)

## Abbreviations

QTL: Quantitative trait loci
SEM: Scanning electron microscope
GBS: Genotype-by-sequencing
Au-Pd: Gold-palladium
HTC: Hard-to-cook
MRDC: Morden Research and Development Centre
dCAPS: Derived cleaved amplified polymorphic sequence
SNP: Single-nucleotide polymorphism
RIL: Recombinant inbred line
BLUEs: Best linear unbiased estimates
META-R: Multi-environment trial analysis with R for windows
RCBD: Randomized complete block design

## References

[CR1] AACC International Approved Methods of Analysis, 11th Ed. Method 56–35.01. Method for Determining Water Hydration Capacity and Percentage of Unhydrated Seeds of Pulses. Approved October 10, 2007. AACC International, St. Paul. doi:10.1094/AACCIntMethod-56-35.01

[CR2] Aguilera JM, Stanley DW (1985). A review of textural defects in cooked reconstituted legumes – the influence of storage and processing. J Food Process Preserv.

[CR3] Akibode CS (2011) Trends in the production, trade, and consumption of food-legume crops in sub-saharan Africa. M.Sc. thesis, Michigan State University, East Lansing. 10 pp

[CR4] Akibode CS, Maredia M (2011) Global and regional trends in production, trade and consumption of food legume crops. Department of Agricultural, Food and Resource Economics, Michigan State University, East Lansing, MI. Report Submitted to SPIA, CGIAR, March 27, 2011

[CR5] Alvarado G, Lopez-Cruz MA, Vargas M, Pacheco A, Rodriguez F, Burgueño J, Crossa J (2015). META-R (multi environment trial analysis with R for windows). Version 5.0.

[CR6] Argel PJ, Paton CJ (1999) Overcoming legume seed hardness. In: Loch DS, Ferguson JE (eds) Forage seed production. CIAT International

[CR7] Bassett MJ (1996). The margo (*mar*) seed coat color gene is a synonym for the joker (*j*) locus in common bean. J Am Soc Hortic Sci.

[CR8] Beninger CW, Hosfield GL (2000). Chemical and morphological expression of the *B* and *Asp* seedcoat genes in *Phaseolus vulgaris*. J Am Soc Hortic Sci.

[CR9] Broman KW, Wu H, Sen Ś, Churchill GA (2003). R/QTL: QTL mapping in experimental crosses. Bioinformatics.

[CR10] Bushey SM, Hosfield GL, Beninger CW (2000). Water uptake and its relationship to pigment leaching in black beans (*Phaseolus vulgaris* L.). Ann Rep Bean Improv Coop.

[CR11] Casañas F, Pérez-Vega E, Almirall A, Plans M, Sabaté J, Ferreira JJ (2013). Mapping of QTL associated with seed chemical content in a RIL population of common bean (*Phaseolus vulgaris* L.). Euphytica.

[CR12] Castellanos JZ, Maldonado HG, Jimenez A, Meija C, Ramos JDJ (1997). Preferential habits of consumers of common bean (*Phaseolus vulgaris* L.) in Mexico. Archivos Latino Americanos de Nutrición, Celaya.

[CR13] Churchill GA, Doerge RW (1994). Empirical threshold values for quantitative trait mapping. Genetics.

[CR14] Cichy KA, Fernandez A, Kilian A, Kelly JD, Galeano CH, Shaw S, Brick M, Hodkinson D, Troxtell E (2014). QTL analysis of canning quality and color retention in black beans (*Phaseolus vulgaris* L.). Mol Breed.

[CR15] Diamant R, Watts BM, Elias LG, Rios B (1989). Consumer utilization and acceptability of raw and cooked black beans (*Phaseolus vulgaris*) in Guatemala. Ecol Food Nutr.

[CR16] Elshire RJ, Glaubitz JC, Sun Q, Poland JA, Kawamoto K, Buckler ES, Mitchell SE (2011). A robust, simple genotyping-bysequencing (GBS) approach for high diversity species. PLoS One.

[CR17] Galeano CH, Fernandez AC, Franco-Herrera N, Cichy KA, McClean PE, Vanderleyden J, Blair MW (2011) Saturation of an intra-gene pool linkage map: towards a unified consensus linkage map for fine mapping and synteny analysis in common bean. PLoS One 6(12). doi:10.1371/journal.pone.0028135 10.1371/journal.pone.0028135 PMC3234260 22174773

[CR18] Galiotou-Panayoutu M, Kyriakidis NB, Margaris I (2007). Phytase-phytate-pectin hypothesis and quality of legumes cooked in calcium solutions. J Sci Food Agric.

[CR19] Glaubitz JC, Casstevens TM, Lu F, Harriman J, Elshire RJ, Sun Q, Bucker ES (2013). TASSEL-GBS: a high capacity genotyping by sequencing analysis pipeline. PLoS One.

[CR20] Kelly JD, Cichy KA, Siddiq M, Uebersax MA (2013). Dry bean breeding and production technologies. Dry beans and pulses: production, processing and nutrition.

[CR21] Kinyanjui PK, Njoroge DM, Makokha AO, Christiaens S, Ndaka DS, Hendrickx M (2015). Hydration properties and texture fingerprints of easy- and hard-to-cook bean varieties. Food Sci Nutr.

[CR22] Konzen ER, Tsai SM (2014). Seed coat shininess in *Phaseolus vulgaris*: rescuing a neglected trait by its screening on commercial lines and landraces. J Agric Sci.

[CR23] Lamprecht H (1940). Zur Genetik von Phaseolus vulgaris. XVII–XVIII. Zwei neue Gene fu¨r Abzeichen auf der Testa, Punc und Mip, sowie u¨ber die Wirkung von V und Inh. Hereditas.

[CR24] Li H, Ye G, Wang J (2007). A modified algorithm for the improvement of composite interval mapping. Genetics.

[CR25] Liu K, Phillips YC, Hung YC, Shewfelt RL, McWatters KH (1992). Hard-cook-defect in cowpeas: storage-induced and treatment induced development. J Food Sci.

[CR26] McWatters KH, Chinnan MS, Worthington RE, Beauchat LR (1987). Influence of storage conditions on quality of cowpea seeds and products processed from stored seeds. J Food Process Preserv.

[CR27] Moghaddam SM, Song Q, Mamidi S, Schmutz J, Lee R, Cregan P, Osorno JM, McClean PE (2014). Developing market class specific InDel markers from next generation sequence data in *Phaseolus vulgaris* L. Front Plant Sci.

[CR28] Neff MM, Neff JD, Chory J, Pepper AE (1998). dCAPS, a simple technique for the genetic analysis of single-nucleotide polymorphisms: experimental applications in *Arabidopsis thaliana* genetics. Plant J.

[CR29] O'Rourke JA, Iniguez LP, Fu F, Bucciarelli B, Miller SS, Jackson SA, McClean PE, Li J, Dai X, Zhao PX, Hernandez G, Vance CP (2014). An RNA-Seq based gene expression atlas of the common bean. BMC Genomics.

[CR30] Pérez-Vega E, Pañeda A, Rodríguez-Suárez C, Campa A, Giraldez R, Ferreira JJ (2010). Mapping of QTLs for morpho-agronomic and seed quality traits in a RIL population of common bean (*Phaseolus vulgaris* L.). Theor Appl Genet.

[CR31] Pérez-Vega E, Campa A, Trabanco N, Casañas F, Giraldez R, Ferreira JJ (2012). Linkage genetic map developed in the Xana/Cornell42492 RIL population: a review. Bean Improv Coop.

[CR32] R Development Core Team (2008) R: a language and environment for statistical computing. R Foundation for Statistical Computing, Vienna, Austria ISBN 3–900051–07-0, URL http://www.R-project.org

[CR33] Ramirez-Cabral NYZ, Kumar L, Taylor S (2016). Crop niche modeling projects major shifts in common bean growing areas. Agr For Meteorol.

[CR34] Schmutz J, McClean PE, Mamidi S, We GA, Cannon SB (2014). A reference genome for common bean and genome-wide analysis of dual domestications. Nat Genet.

[CR35] van Schoonhoven A, Pastor-Corrales MA (1987). Standard system for the evaluation of bean germplasm.

[CR36] Shiga TM, Lajolo FM, Filisetti CC (2004). Changes in the cell wall polysaccharides during storage and hardening of beans. Food Chem.

[CR37] Stanley DW (1992). Hard beans-a problem for growers, processors, and consumers. Hort Technol.

[CR38] Statistics Canada (2016) CANSIM table 001–0010, Estimated areas, yield, production and average farm price of principal field crops, in metric units annual (Accessed 19 Sept 2017)

[CR39] Van Os H, Stam P, Visser RGF, Van Eck HJ (2005). RECORD: a novel method for ordering loci on a genetic map. Theor Appl Genet.

[CR40] Voorrips RE (2002). MapChart: software for the graphical presentation of linkage maps and QTLs. J Hered.

