# Supplementary material for: Genetic analysis and QTL mapping of the seed hardness trait in a black common bean (Phaseolus vulgaris) recombinant inbred line (RIL) population
Source: Mol Breed. 2018 Feb 23;38(3):34. doi: 10.1007/s11032-018-0789-y (PMC5842266; doi:10.1007/s11032-018-0789-y)
Supplement: Supplementary file 1 — (DOCX 213 kb) [file 11032_2018_789_MOESM1_ESM.docx]

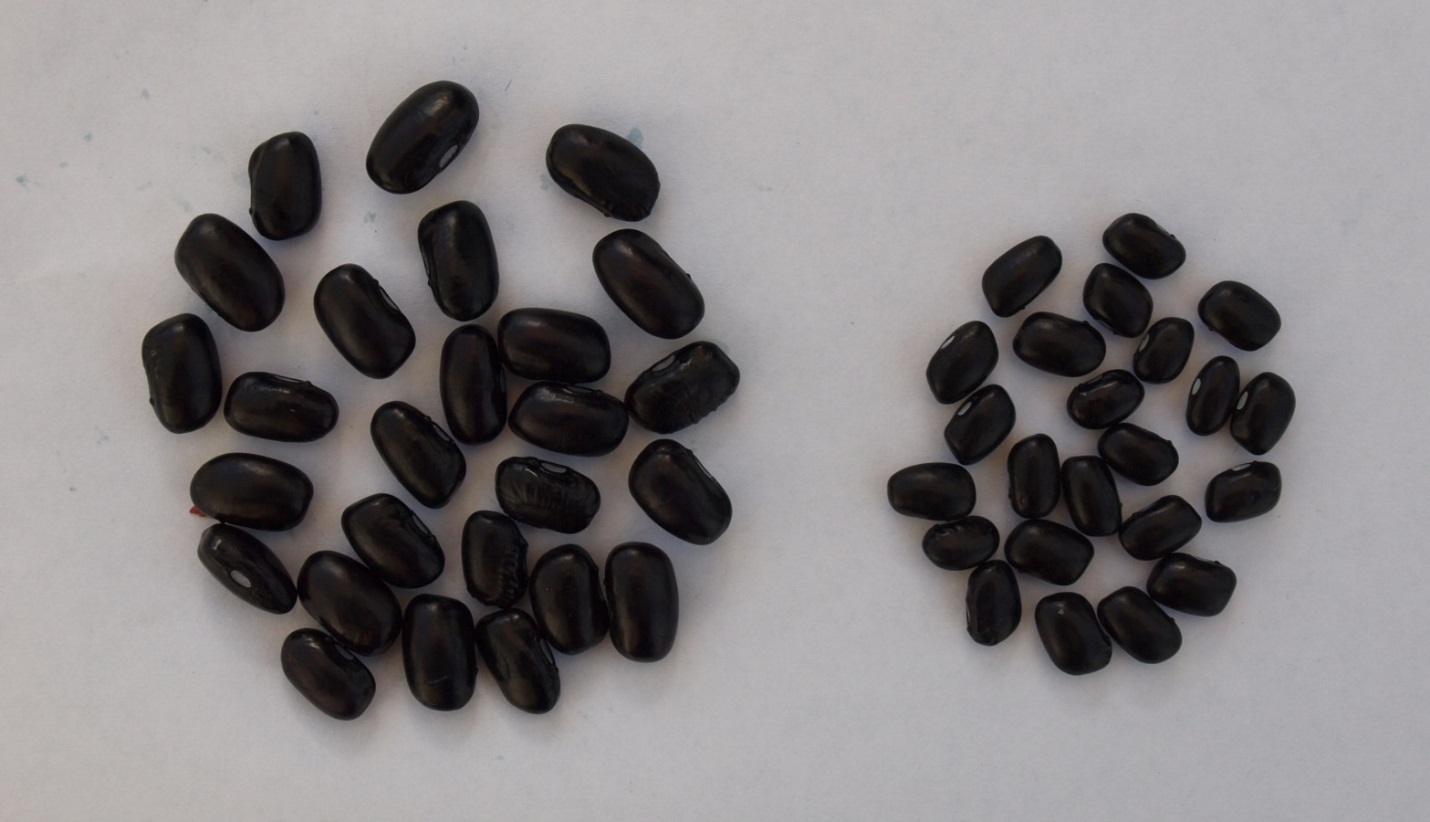


(b)

(a)

**Supplementary Fig. 1** Hydrated seeds (a) and stone seeds (b) in a sample of black bean seeds after 16 h soaking in water at 22°C.
