# Supplementary material for: Genetic analysis and QTL mapping of the seed hardness trait in a black common bean (Phaseolus vulgaris) recombinant inbred line (RIL) population
Source: Mol Breed. 2018 Feb 23;38(3):34. doi: 10.1007/s11032-018-0789-y (PMC5842266; doi:10.1007/s11032-018-0789-y)
Supplement: Supplementary file 2 — (DOCX 884 kb) [file 11032_2018_789_MOESM2_ESM.docx]

**Supplementary Fig. 2** QTL effects (a-d) and interactions (e-h) estimated from the second-harvest seeds at Carman site in 2016. Error bars indicate SE.


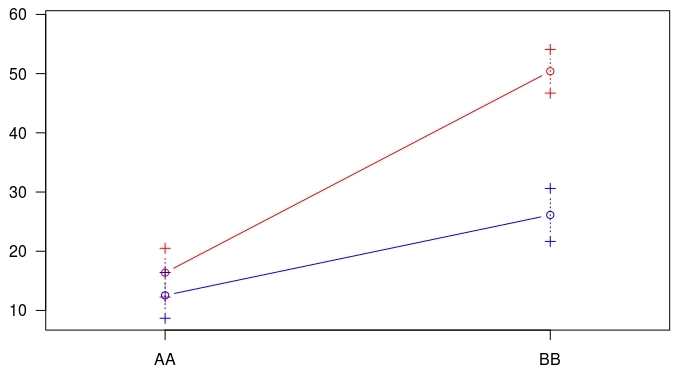


*Asp*

Interaction plot for BM2c0024 and *Asp*

(f)


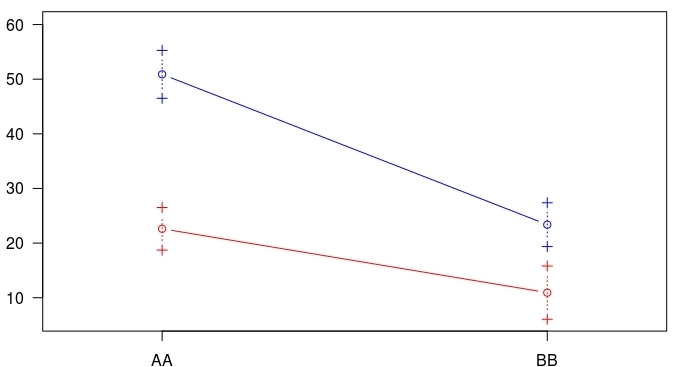


BM02c0024

Interaction plot for dCAPS01c5176 and BM02C0024

(g)


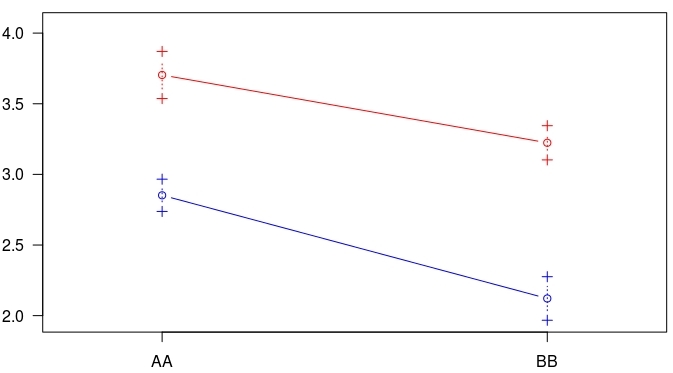


*Asp*

Interaction plot for BM04c0163 and *Asp*

(h)

SSP


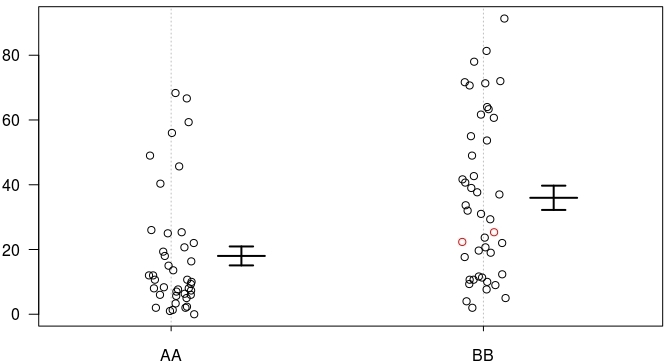


dCAPS01c5176

(b)

SSP


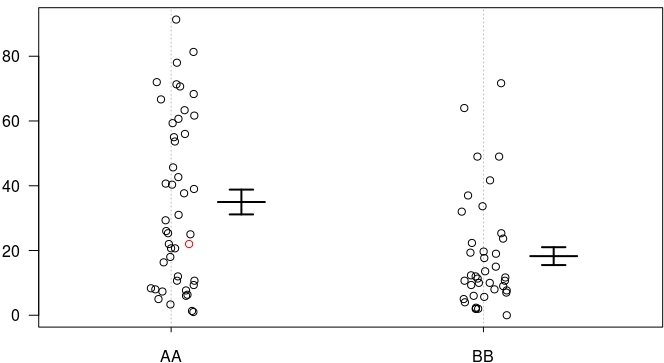


BM02c0024

(c)

VSC


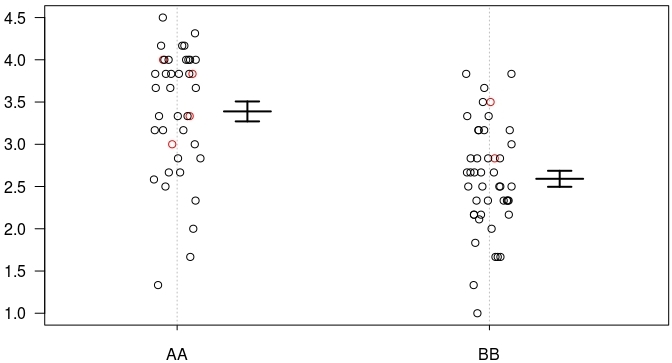


BM04c0163

(d)

SSP


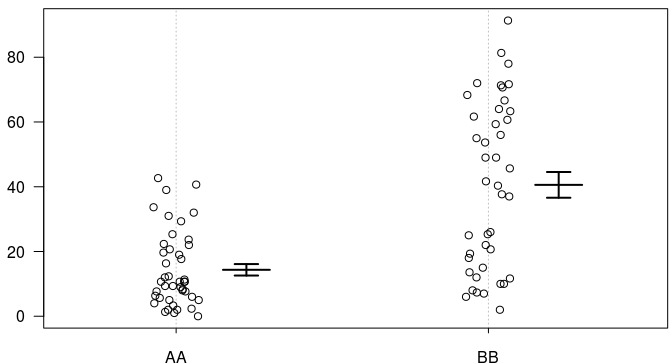


*Asp*

BK (AA)

H68 (BB)

(a)


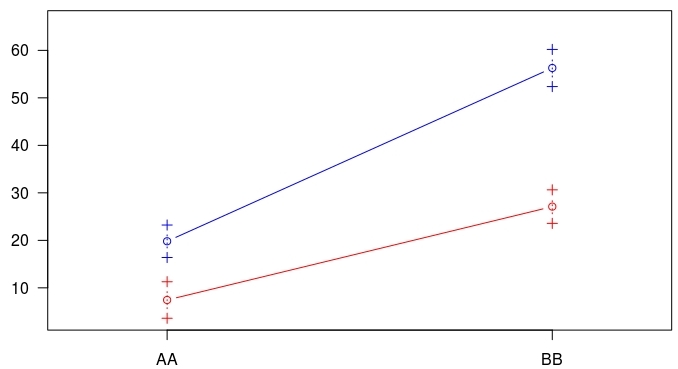


ASP

Interaction plot for dCAPS01c5176 and *Asp*

(e)

BK (AA)

H68 (BB)
