# Supplementary material for: Genetic analysis and QTL mapping of the seed hardness trait in a black common bean (Phaseolus vulgaris) recombinant inbred line (RIL) population
Source: Mol Breed. 2018 Feb 23;38(3):34. doi: 10.1007/s11032-018-0789-y (PMC5842266; doi:10.1007/s11032-018-0789-y)
Supplement: Supplementary file 3 — (DOCX 5410 kb) [file 11032_2018_789_MOESM3_ESM.docx]

(d)

(c)

(b)

(a)


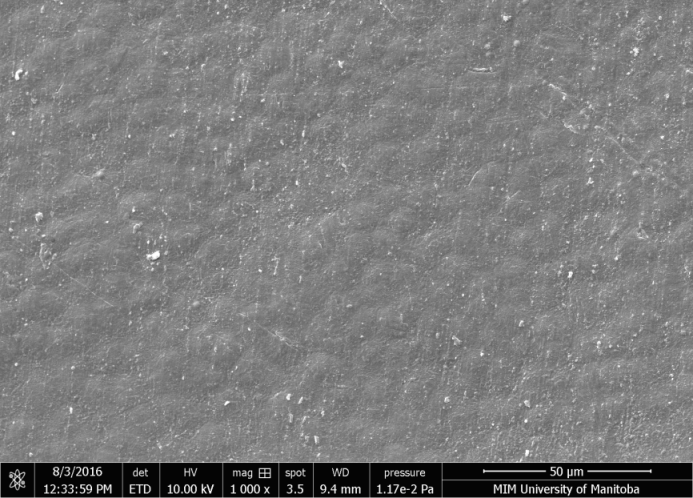

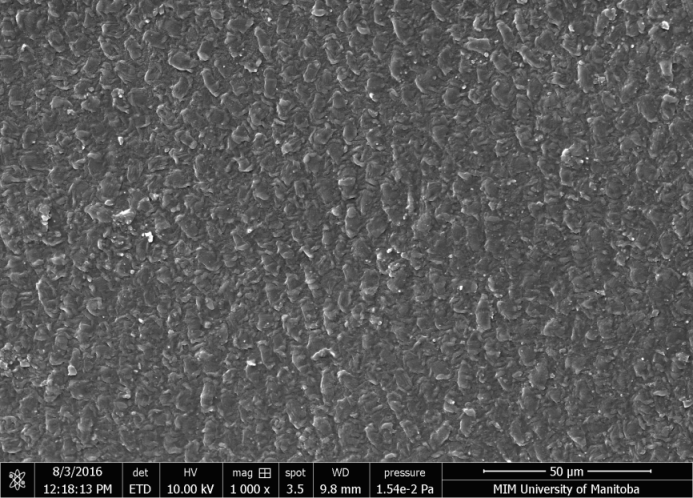

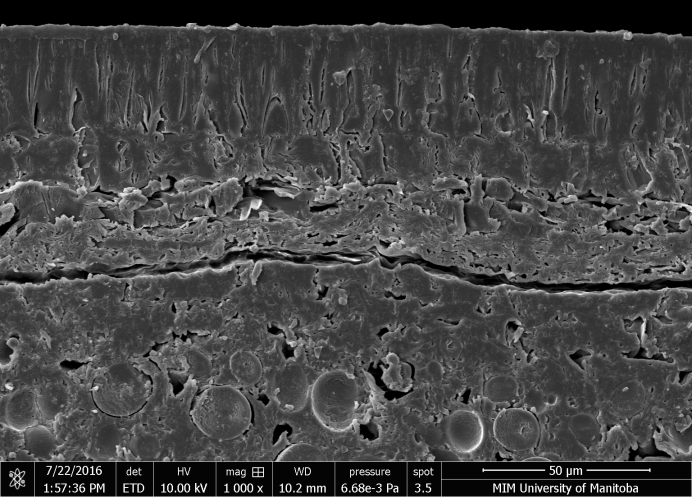

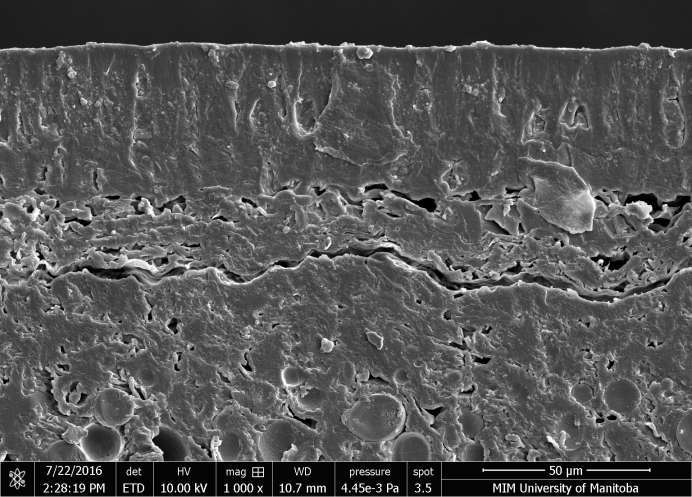


**Supplementary Fig. 3** Scanning Electron Microscope images of seed coat surface and cross-sections of H68-4 (a, and b) and BK004-001 (c, and d). Seed samples were collected from the field during 2015-2016. The seed was sectioned using a sharp glass blade and observed using a Quanta 650 FEG Environmental Scanning Electron Microscope at the Engineering Department, University of Manitoba.
