# Supplementary material for: Genetic analysis and QTL mapping of the seed hardness trait in a black common bean (Phaseolus vulgaris) recombinant inbred line (RIL) population
Source: Mol Breed. 2018 Feb 23;38(3):34. doi: 10.1007/s11032-018-0789-y (PMC5842266; doi:10.1007/s11032-018-0789-y)
Supplement: Supplementary file 4 — (DOCX 15 kb) [file 11032_2018_789_MOESM4_ESM.docx]

**Supplementary Fig.** **4** Coding sequence of PHAVU_001G264400g showing the amino acid differences between BK04-001 and H68-4. Two threonines in BK04-001 are replaced by a single asparagine in H68-4.

Gene Name= PHAVU_001G264400g

Sequence ID: XM_007163729.1

>BK04-001_Pv_Cystatin_CDS

ATGGCTGCGACTCTCGCCATTTTCGTCACCTTGCTCTCGCTTCTCTCTTCTGCTTCATGTGCTCGATTGGTCGGGGGGAAGACGGAGATCCCTGATGTCAGAACAAACAGGGAAGTGCAAGAGCTTGGGAGGTTCTCGGTGGAGGAGTACAACAACGGTTTGAAGCTCTACAACGACAGCGATAACGAGAAGTTGACCTTTTCAGAGGTGGTTGAGGCGCAACAACAAGTGGTGTCAGGGGTGAAGTACTACTTGAAGATCTCTGCTACTCACAGAGGGATTCACAAAATGTTCTCCTCAGTGGTGGTGGTCAAGCCCTGGATTCATTCCAAGAAGCTCCTCCACTTTTCCCCTGCATCCACCACCACCACCACCACCAACAACAACCAGTGA

>ProteinSeq_BK04-001

MAATLAIFVTLLSLLSSASCARLVGGKTEIPDVRTNREVQELGRFSVEEYNNGLKLYNDS

DNEKLTFSEVVEAQQQVVSGVKYYLKISATHRGIHKMFSSVVVVKPWIHSKKLLHFSPAS

TTTTTTNNNQ

>H68-4_Pv_Cystatin_CDS

ATGGCTGCGACTCTCGCCATTTTCGTCACCTTGCTCTCGCTTCTCTCTTCTGCTTCATGTGCTCGATTGGTCGGGGGGAAGACGGAGATCCCTGATGTCAGAACAAACAGGGAAGTGCAAGAGCTTGGGAGGTTCTCGGTGGAGGAGTACAACAACGGTTTGAAGCTCTACAACGACAGCGATAACGAGAAGTTGACCTTTTCAGAGGTGGTTGAGGCGCAACAACAAGTGGTGTCAGGGGTGAAGTACTACTTGAAGATCTCTGCTACTCACAGAGGGATTCACAAAATGTTCTCCTCAGTGGTGGTGGTCAAGCCCTGGATTCATTCCAAGAAGCTCCTCCACTTTTCCCCTGCATCCACCACCACCACCAACAACAACAACCAGTGA

>ProteinSeq_H68-4

MAATLAIFVTLLSLLSSASCARLVGGKTEIPDVRTNREVQELGRFSVEEYNNGLKLYNDS

DNEKLTFSEVVEAQQQVVSGVKYYLKISATHRGIHKMFSSVVVVKPWIHSKKLLHFSPAS

TTTTNNNNQ
